# Telomerase Reverse Transcriptase (TERT) is a Therapeutic Target of Oleanane Triterpenoid CDDO-Me in Prostate Cancer

**DOI:** 10.3390/molecules171214795

**Published:** 2012-12-11

**Authors:** Yongbo Liu, Xiaohua Gao, Dorrah Deeb, Ali S. Arbab, Subhash C. Gautam

**Affiliations:** 1 Department of General Surgery, Henry Ford Health System, Detroit, MI 48202, USA; E-Mails: bliu1@hfhs.org (B.L.); tgao1@hfhs.org (X.G.); ddeeb1@hfhs.org (D.D.); 2 Department of Radiology, Henry Ford Health System, Detroit, MI 48202, USA; E-Mail: sali1@hfhs.org

**Keywords:** CDDO-Me, hTERT, telomerase activity, apoptosis, prostate cancer

## Abstract

Methyl-2-cyano-3,12-dioxooleana-1,9(11)-dien-28-oate (CDDO-Me) is an synthetic oleanane triterpenoid with strong antiprolifertive and proapoptotic activities in cancer cells. However, the effect of CDDO-Me on human telomerase reverse transcriptase (hTERT) and its telomerase activity in prostate cancer cells has not been studied. We investigated the role of hTERT in mediating the anticancer activity of CDDO-Me in prostate cancer cells *in vitro* and *in vivo*. The inhibition of cell proliferation and induction of apoptosis by CDDO-Me in LNCaP and PC-3 prostate cancer cell lines was associated with the inhibition of hTERT gene expression, hTERT telomerase activity and a number of proteins that regulate hTERT transcriptionally and post-translationally. Furthermore, ablation of hTERT protein increased the sensitivity of cancer cells to CDDO-Me, whereas its overexpression rendered them resistant to CDDO-Me. In addition, inhibition of progression of preneoplastic lesions (*i.e.*, low and high-grade prostate intraepithelial neoplasms, PINs) to adenocarcinoma of the prostate by CDDO-Me in TRAMP mice was associated with significant decrease in TERT and its regulatory proteins in the prostate gland. These data provide evidence that telomerase is a potential target of CDDO-Me for the prevention and treatment of prostate cancer.

## 1. Introduction

2-Cyano-3,12-dioxooleana-1,9(11)-dien-28-oic acid (CDDO) and its C-28 imidazole [1-(2-cyano-3,12-dioxooleana-1,9-dien-28-oyl) imidazole (CDDO-Im)] and C-28 methyl ester [methyl-2-cyano-3,12-dioxooleana-1,9(11)-dien-28-oate (CDDO-Me)] derivatives are multifunctional synthetic oleanane triterpenoids with potent antitumorigenic properties [1]. CDDOs inhibit proliferation and induce apoptosis in diverse types of tumor cell lines [2,3,4]. The anticancer mechanisms of CDDOs include induction of apoptosis and modulation of several signal transduction pathways, such as PI3K/Akt/mTOR, MAPK (Erk1/2), NF-κB, TGF-β/Smad and Nrf2 signaling pathways [5,6,7]. However, the effect of CDDOs on telomerase, the reverse transcriptase that elongates telomeres has not been adequately studied. Telomeres are nucleoprotein structures present at the end of chromosomes, which are essential in maintaining chromosome stability by preventing end-to-end fusion and chromosomal rearrangement [8]. During each cell division, telomere length is progressively shortened due to gradual loss of telomeric DNA sequence (TTAGGG) [9,10]. Eventually, shortening of telomeres beyond a critical threshold leads to replicative senescence or apoptosis. Telomerase maintains telomere length by adding the hexameric DNA repeats (TTAGGG) to the 3' flanking end of DNA strands in telomeres. The human telomerase complex consists of telomerase reverse transcriptase (hTERT), telomerase RNA (TERC), telomerase associated protein-1 (TEP-1), hsp90 and p23 [11,12,13]. In humans telomerase activity is repressed in normal somatic cells but is detectable in germ line cells and certain stem cells [14,15]. Deregulated telomerase activity is associated with promotion of tumorigenesis and neoplastic growth of cancers [16,17]. In fact, approximately 85 to 90% of human cancers exhibit reactivation of telomerase activity [18]. Thus, telomerase inhibition in cancer cells is an attractive target for developing novel anticancer therapeutics. Indeed, we have recently shown that the inhibition of cell proliferation and induction of apoptosis by CDDO-Me in pancreatic cancer cells was associated with the inhibition of telomerase activity [19]. We have also reported that CDDOs inhibit the progression of the carcinoma of the prostate (CaP) in the TRAMP mouse model of prostate cancer through the inhibition of antiapoptotic Akt and NF-κB signaling proteins [20,21]. Since Akt and NF-κB play a critical role in the posttranslational regulation of hTERT telomerase activity and nuclear translocation [22,23], in the present study we investigated whether the antiproliferative and apoptosis-inducing effects of CDDO-Me in prostate cancer cells correlate with the inhibition of telomerase activity.

## 2. Results and Discussion

### 2.1. CDDO-Me Inhibits Proliferation and Induces Apoptosis in Prostate Cancer Cells

**Figure 1:**
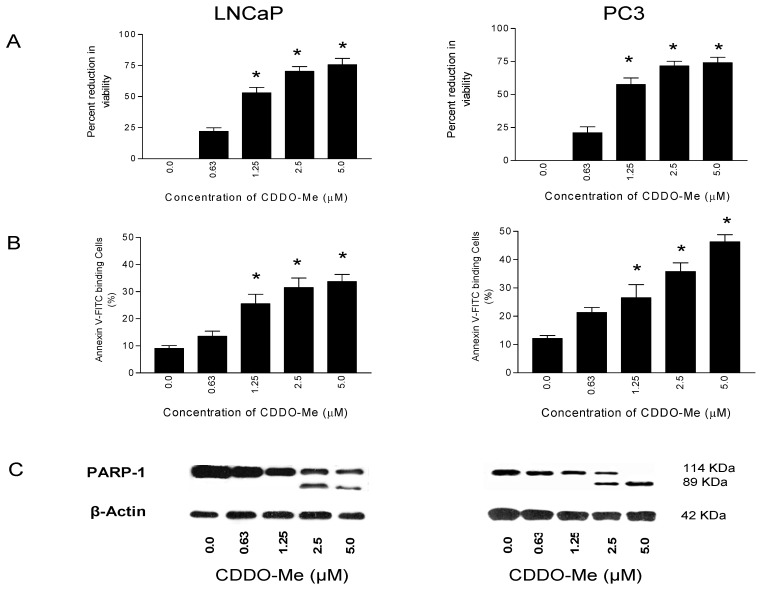
CDDO-Me inhibits proliferation and induces apoptosis in prostate cancer cells. (**A**) LNCaP and PC-3 cells (1 × 10^4^/well) were treated with CDDO-Me at concentrations ranging from 0 to 5 µM for 72 h in a 96-well microtiter plate in triplicates. Cell viability was measured by MTS assay using CellTiter AQueous Assay System. (**B**) Annexin V-FITC binding. Tumor cells were treated with CDDO-Me at 0 to 5 µM for 24 h and then reacted with 5 μL of annexin V-FITC and 5 μL PI for 30 min and the percentage of annexin V-FITC binding cells was determined by flow cytometry. (**C**) Cleavage of PARP-1 in cells treated with CDDO-Me was analyzed by immunoblotting. Similar results were obtained in 3 independent experiments. * *p* < 0.01 compared to control cells (no CDDO-Me).

The effect of CDDO-Me on the proliferation of LNCaP and PC-3 human prostate cancer cell lines was measured by an MTS assay. As shown in Figure 1A, treatment with CDDO-Me for 72 h at a concentration of 0.63 µM had minimal growth inhibition (~21%). However, at concentrations of 1.25 to 5 µM the growth inhibitory effect of CDDO-Me was significantly higher (LNCaP, 53 to 76% inhibition, *p* < 0.01; PC-3, 57% to 74% inhibition, *p* < 0.01). Whether inhibition of cell proliferation by CDDO-Me was associated with induction of apoptosis was determined by the binding of annexin V-FITC to cells treated with CDDO-Me. For this, tumor cells were treated with CDDO-Me at concentrations of 0 to 5 µM for 24 h and then treated with annexin V-FITC and propidium iodide and analyzed by flow cytometry. As shown in Figure 1B, a small percentage of untreated LNCaP and PC-3 bound annexin V-FITC (9% and 12%, respectively). The percentage of annexin V-FITC binding LNCaP cells treated with CDDO-Me increased in concentration-dependent manner from 14% at 0.63 to 34% at 5 µM CDDO-Me compared to untreated cells (*p* < 0.01). The percentage of annexin V-binding PC-3 cells also increased from 21% to 46% following treatment with CDDO-Me (0.063–5 µM). Induction of apoptosis by CDDO-Me was confirmed by the cleavage of native PARP-1 (114 kDa) and the appearance of the cleaved PARP-1 fragment (89 kDa) in both cell lines treated with CDDO-Me. Figure 1C shows the appearance of a 89 kDa cleaved PARP-1 fragment at 2.5 and 5 μM; however, in some experiments the cleavage product was also seen at 1.25 μM CDDO-Me. Collectively, these data demonstrated that CDDO-Me at concentrations of 0.063 to 5 µM inhibits proliferation and induces apoptosis in prostate cancer cells.

### 2.2. CDDO-Me Inhibits hTERT Expression and Telomerase Activity in Prostate Cancer Cells

**Figure 2:**
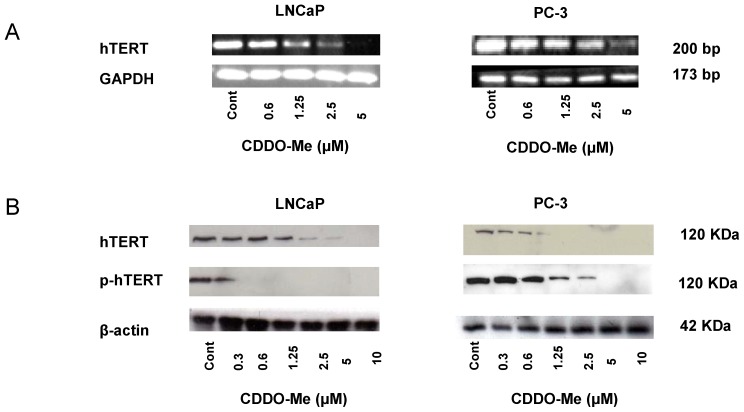
CDDO-Me inhibits hTERT gene and hTERT protein expression. (**A**) Effect of CDDO-Me on hTERT mRNA expression. (**B**) Shows effect of CDDO-Me on basal and p-hTERT protein. Each experiment was repeated at least two times.

Since tumor cells have active telomerase which promotes tumor growth by modulating the expression of growth controlling genes and enhancing cell proliferation [24], we determined the effect of CDDO-Me on the expression of hTERT and its telomerase activity in LNCaP and PC-3 cells. For this, we analyzed hTERT mRNA by RT-PCR and hTERT protein by Western blotting. Treatment with CDDO-Me at concentrations of 1.25 to 5 μM significantly reduced hTERT mRNA in both cell lines without affeting GAPDH mRNA (Figure 2A). CDDO-Me also sharply reduced hTERT protein levels at 0.63 to 5 μM CDDO-Me (Figure 2B). Since phosphorylation of the catalytic subunit of hTERT is necessary for telomerase activity and nuclear translocation, we measured the effect of CDDO-Me on p-hTERT (ser^826^). As can be seen in Figure 2B, CDDO-Me inhibited both hTERT and p-hTERT at concentrations of 0.63 μM and above in both cell lines.

**Figure 3:**
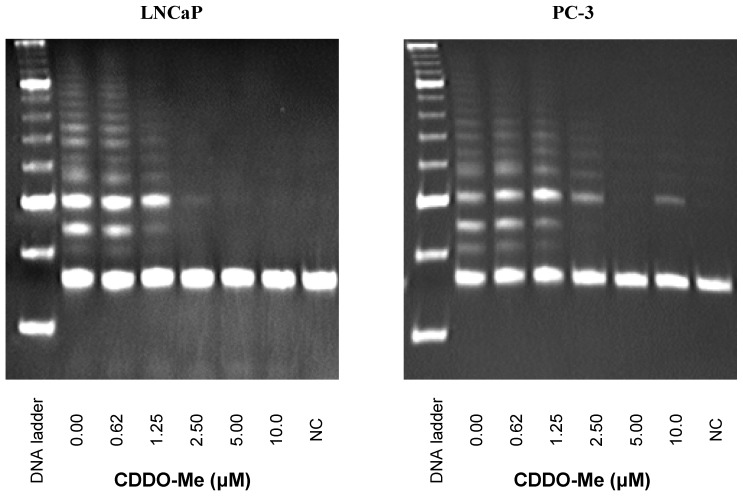
CDDO-Me inhibits telomerase activity. LNCaP and PC-3 cells were treated with CDDO-Me (0–10 μM) for 48 h and telomerase activity in cell extracts was measured by TRAP assay as described in Materials and Methods. Gels show DNA laddering patterns under different treatment conditions. NC, negative control (no cell extract). The DNA ladder is 10 bp.

Whether CDDO-Me affects the telomerase activity of hTERT was determined next. LNCaP and PC-3 cells were treated with CDDO-Me (0–10 μM) for 48 h and cells were extracted in CHAP lysis buffer. The telomerase activity of extracts was measured using the PCR-based TRAP assay. While there was ~30% reduction in the telomerase activity in both cell lines treated with 0.63 μM CDDO-Me, but it was sharply reduced or completely abolished in cells treated with CDDO-Me at 1.25 μM and above as identified by significant reduction or complete loss of DNA laddering (Figure 3).

### 2.3. CDDO-Me Inhibits hTERT Regulatory Proteins

**Figure 4:**
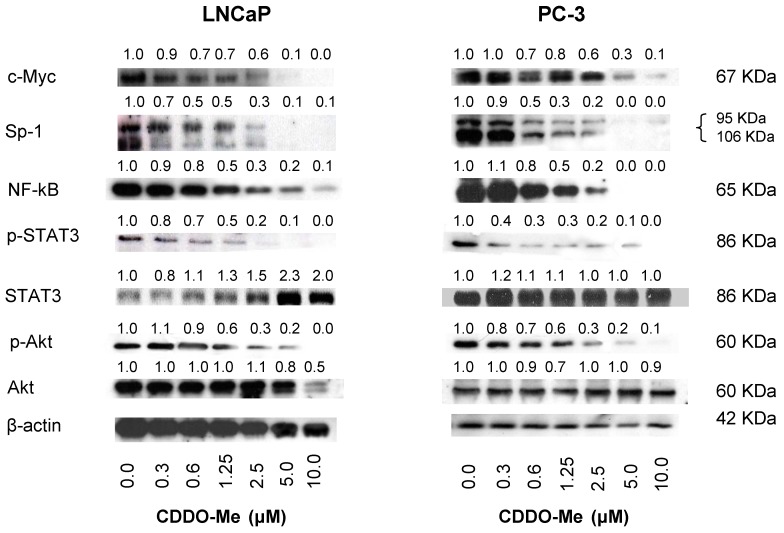
CDDO-Me inhibits hTERT regulatory proteins. LNCaP and PC-3 cells were treated with CDDO-Me (0–5 μM) for 48 h and cell lysates were analyzed for c-Myc, Sp1, NF-κB, STAT-3, p-STAT-3, Akt and p-Akt by western blotting. Numbers on top of each blot represent signal strengths relative to the signal strength of untreated control samples considered as 1.

Several regulatory molecules that regulate hTERT expression and telomerase activity have been identified. The hTERT core promoter contains several transcription factor binding sites including those of c-Myc, Sp1, NF-κB and STAT-3 [25,26,27,28]. Therefore, we assessed the effect of CDDO-Me on the levels of these proteins. Treatment with CDDO-Me (0 to 5 μM) for 48 h significantly reduced the levels of c-Myc, Sp1 and NF-κB in a dose-dependent manner, whereas it only affected the phosphorylation of STAT-3 (Figure 4). Post-translationally, Akt phosphorylates hTERT on Ser^227^ and Ser^826^ activating its telomerase activity and nuclear translocation [22,23]. CDDO-Me inhibited p-Akt (Ser^826^) levels in both cell lines in a concentration-dependent manner (Figure 4) and reduced basal Akt in LNCaP cells at 10 μM. These data suggested that inhibition of c-Myc, Sp1, NF-κB, p-STAT-3 and p-Akt contributes to the down-regulation of hTERT transcriptionally and post-translationally.

### 2.4. hTERT is a Target of CDDO-Me

**Figure 5:**
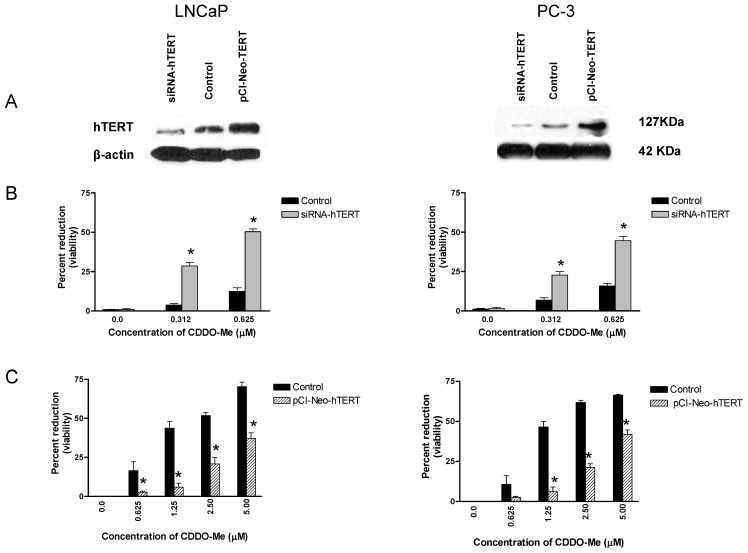
hTERT regulates response to CDDO-Me. LNCaP and PC-3 cells were transfected with siRNA-hTERT or hTERT overexpression plasmid (pCI-neo-hTERT) for 48 h. hTERT levels were measured by immunoblotting (**A**) and response to CDDO-Me was assessed in 72 h MTS assay (**B** and **C**). Each experiment was repeated two times. * *p* < 0.05 compared to control cells.

To more directly demonstrate the involvement of hTERT in mediating the antiproliferative and apoptosis-inducing activity of CDDO-Me, we genetically altered the expression of hTERT in tumor cells and measured their response to CDDO-Me. For this, LNCaP and PC-3 cells were transfected with hTERT siRNA or hTERT overexpression plasmid for 48 h to decrease or increase hTERT expression level. As shown in Figure 5A, transfection with siRNA-hTERT decreased, whereas transfection with hTERT expression plasmid increased the level of hTERT in both cell lines as detected by Western blotting. Decrease in hTERT levels increased the susceptibility of both cell lines to concentrations of CDDO-Me which are otherwise inactive or only slightly active (*i.e.*, 0.312–0.625 μM, Figure 5B). On the other hand, overexpression of hTERT significantly reduced the sensitivity of both cell lines to CDDO-Me at concentrations of 0.625 to 5 μM (Figure 5C, *p* < 0.05). Transfection with non-targeting siRNA or empty plasmid had no effect on response of cells to CDDO-Me (data not shown). These data demonstrated that hTERT is a molecular target of CDDO-Me in prostate cancer cells.

### 2.5. Inhibition of PINs by CDDO-Me in TRAMP Mice is Associated with Inhibition of hTERT

**Figure 6:**
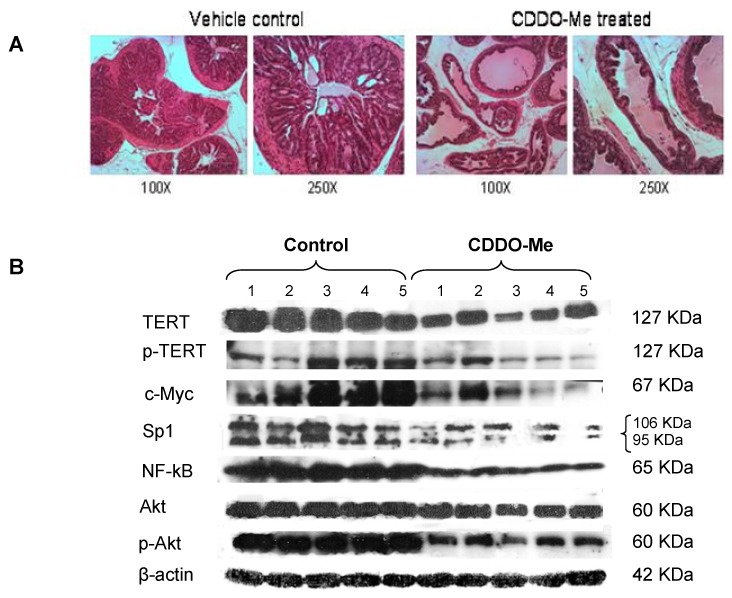
Effect of CDDO-Me on prostate cancer progression, TERT and TERT regulatory proteins in DLP of TRAMP mice. (**A**) H&E stained sections of DLP showing inhibition of progression of PINs to adenocarcinoma of the prostate in a mouse treated with CDDO-Me for 20 weeks (n = 10). (**B**) Immunoblots showing effect of CDDO-Me on levels of TERT, p-TERT, c-Myc, Sp1, NF-κB, Akt and p-Akt in DLP of nontreated (vehicle control) and CDDO-Me treated mice (5 each).

We have previously shown that administration of CDDO-Me to TRAMP mice starting at 5 weeks of age at a dose of 15 μmol/kg/day, 5 days/wk by oral gavage for 20 weeks resulted in inhibition of progression of preneoplastic lesions (low and high-grade PINs) to adenocarcinoma of the prostate in more than 70% of the mice without noticeable toxicity [20,21]. Treatment with CDDO-Me also inhibited metastasis of cancer to lung, liver, kidney and pelvic lymph nodes. As shown in Figure 6A, dorso-lateral prostate lobe (DLP) of a 25 weeks old control mouse exhibited well-differentiated adenocarcinoma of the prostate, whereas most of the glands in CDDO-Me treated mice were normal or have low-grade PIN lesions. To determine whether inhibition of PINs by CDDO-Me was associated with the inhibition/decrease in TERT and its regulatory proteins, DLP tissue was analyzed for TERT, p-TERT, c-Myc, Sp1, NF-κB, Akt and p-Akt. Figure 6B shows the levels of these proteins in DLP of nontreated (control) and CDDO-treated mice (five each).

Treatment with CDDO-Me reduced the levels of TERT and p-TERT in the DLP of most of mice (38% and 42% reduction, respectively). c-Myc, Sp1 and NF-κB were also reduced (51%, 60% and 68% reduction, respectively). On the other hand, there was only a slight reduction in Akt in the DLP of treated mice but Akt phosphorylation was significantly reduced (48% reduction). These data indicated that response to CDDO-Me in TRAMP mice was associated with reduction in proteins that regulate production and phosphorylation of TERT.

### 2.6. Discussion

More than ninety percent of all human cancers exhibit reactivation of telomerase activity [18]. Activated telomerase promotes tumor growth by modulating the expression of growth controlling genes and enhancing cell proliferation [24]. On the other hand, inhibition of telomerase activity in tumor cells induces telomere shortening and apoptosis [29]. Thus, the antiproliferative and apoptosis inducing effects of CDDO-Me in cancer cells could be mediated at least in part through the inhibition of telomerase activity. Indeed, the present study showed that inhibition of cell proliferation and induction of apoptosis in prostate cancer cells by CDDO-Me was associated with the inhibition of hTERT and its telomerase activity.

The inhibition of telomerase by CDDO-Me could be attributed to the inhibition of hTERT production and/or its phosphorylation. Analysis of hTERT mRNA by RT-PCR showed that CDDO-Me attenuated hTERT mRNA. Further, Western blot analysis of cellular proteins revealed that CDDO-Me also decreased the levels of both basal and phosphorylated hTERT. In addition, CDDO-Me also significantly inhibited the hTERT telomerase activity as measured by TRAP assay. Together, attenuation of hTERT gene expression, protein phosphorylation and telomerase activity by CDDO-Me indicated that inhibition hTERT expression and its telomerase activity are part of the mechanism by which CDDO-Me inhibits proliferation and induce apoptosis in prostate cancer cells. These findings are in agreement with other reports showing that inhibition of hTERT telomerase activity is necessary for the antiproliferative and apoptosis-inducing activity of compounds such as genistein, sulforaphane and curcumin [30,31]. However, more work is required to determine whether inhibition of hTERT by CDDO-Me also leads to shortening of telomeres and whether CDDO-Me directly binds and degrades RNA template of telomerase.

A number of transcription factors and molecules that regulate hTERT expression and telomerase activity have been identified. The hTERT core promoter contains several transcription factor binding sites including binding sites for c-Myc, Sp1, NF-κB and STAT-3 [25,26,27,28]. Inhibition of these transcription factors would likely impact transcription of hTERT gene. Indeed, we found that CDDO-Me inhibited c-Myc, Sp1, NF-κB and STAT-3 in LNCaP and PC-3 cells, indicating that diminished hTERT expression and protein production by CDDO-Me may be attributed to the inhibition of these transcription factors. The role of Sp1 in transcription of hTERT gene has been well-documented. Sp1 interacts with c-Myc to enhance hTERT expression [25,26]. On the other hand, the role of Sp3 and Sp4 in hTERT expression has not been investigated. Sp transcription factors (Sp1, Sp3 and Sp4) are overexpressed in most cancer cells including prostate cancer cells and many genes that play a critical role in tumorigenesis (e.g., cyclin D1, survivin and VEGF) are regulated by Sp transcription factors [32,33,34,35]. Sp factors are targets of CDDO-Me like compounds such as betulinic acid and its synthetic derivatives; therefore, it is not surprising that CDDO-Me inhibited Sp1 and other transcription factors that control hTERT expression. However, the role of Sp3 and Sp4 in transcription of hTERT remains to be determined.

Post-translationally, phosphorylation of hTERT on Ser^227^ and Ser^826^ by Akt is required for its activation and nuclear translocation. Thus, in addition to reduced hTERT expression, the inhibition of telomerase activity of hTERT could also be attributed to the inhibition hTERT phosphorylation by Akt. Indeed, our data showed that CDDO-Me inhibited phosphorylation (activation) of Akt without significantly affecting basal Akt. We have previously shown that CDDO-Me specifically inhibits Akt kinase activity without affecting the activity of PDK1, the upstream kinase that phosphorylates and activates Akt or Akt specific phosphatases [36]. Others have shown that inhibition of Sp factors also decreases phosphorylation of Akt and expression of c-Myc and NF-κB [35,37,38]; therefore, the possibility exists that CDDO-Me could inhibit p-Akt, c-Myc and NF-κB indirectly through the inhibition of Sp transcription factors. In any event, the inhibition of Akt phosphorylation and NF-κB by CDDO-Me would impair nuclear accumulation of hTERT since phosphorylated hTERT must associate with Akt and NF-κB for nuclear translocation [22,23]. hTERT is also regulated epigenetically through histone acetylation and DNA methylation at the promoter sites [39]. Whether CDDO-Me impacts these epigenetic events remains to be determined.

The present study also demonstrated the relevance of hTERT in mediating the antitumor activity of CDDO-Me in prostate cancer cells as cellular level of hTERT influenced the response to CDDO-Me. We found that knocking-down the expression of hTERT by siRNA hTERT increased the susceptibility of LNCaP and PC-3 cells to concentrations of CDDO-Me which are otherwise inactive or only slightly active (*i.e.*, 0.312–0.625 μM, *p* < 0.05). On the other hand, increasing hTERT level by tranfecting cells with hTERT overexpression plasmids significantly reduced the susceptibility of cells to CDDO-Me at concentrations that significantly inhibited the proliferation of these cells (e.g., 0.625–5 μM, *p* < 0.05). These data suggested that hTERT is a potential molecular target of CDDO-Me in prostate cancer cells.

Cancer-specific activation of telomerase makes it an attractive target for therapeutic intervention. Earlier we have shown efficacy of CDDO and CDDO-Me in preventing the progression of preneoplastic lesions to adenocarcinoma of the prostate in TRAMP mice [20,21]. Protein analysis of prostate tissue showed that treatment with CDDO-Me inhibited TERT production and phosphorylation in DLP. In addition, CDDO-Me also reduced the levels of c-Myc and Sp1 which regulate the transcription of TERT and that of p-Akt and NF-κB which regulate hTERT/telomerase post-translationally. Thus, association between the inhibition of cancer progression and inhibition of p-TERT and other regulatory proteins indicates that TERT is involved in mediating the antitumor activity of CDDO-Me in TRAMP mice.

## 3. Experimental

### 3.1. Reagents

CDDO-Me was obtained from the National Cancer Institute (Bethesda, MD, USA) through the Rapid Access to Intervention Development Program. Antibodies against Akt, p-Akt (ser^473^), NF-κB (p65), STAT3, p-STAT3 (ser^736^), Sp1, c-Myc and β-actin were purchased from Santa Cruz Biotechnology, Inc. (Santa Cruz, CA, USA). Anti-hTERT and p-TERT antibodies were obtained from Abcam Inc. (Cambridge, MA, USA). CellTiter 96® AQueous One Solution Proliferation Assay System was from Promega (Madison, WI, USA). Annexin V-FITC apoptosis detection kit II was obtained from BD Pharmingen (San Diego, CA, USA) and TRAPeze telomerase detection kit was purchased from Millipore (Millipore, Temecula, CA, USA).

### 3.2. Cell Culture

Prostate cancer cell lines LNCaP and PC-3 were obtained from the American Type Culture Collection (ATCC, Rockville, MD, USA). LNCaP cells were cultured in RPMI-1640 medium whereas PC-3 cells were cultured in F-12K medium supplemented with 10% FBS, 1% penicillin/streptomycin, and 25 mM HEPES buffer.

### 3.3. MTS Assay

1 × 10^4^ cells in 100 μL of cell culture medium were seeded into each well of a 96-well plate. After incubation for 24 h, cells were treated with CDDO-Me for 72 h. Cell viability was then determined by the colorimetric MTS assay using the CellTiter 96 AQueous One Solution Proliferation Assay System from Promega following the instructions provided by the manufacturer.

### 3.4. Analysis of Apoptosis

Induction of apoptosis by CDDO-Me was assessed by the binding of annexin V to phosphatidylserine, which is externalized to the outer leaflet of the plasma membrane early during induction of apoptosis and from the cleavage of PARP-1 by Western blotting. For annexin V-FITC binding, tumor cells were treated with CDDO-Me for 24 h, harvested and resuspended in the binding buffer provided in the annexin V-FITC apoptosis detection kit. Cells were reacted with 5 μL of annexin V-FITC reagent and 5 μL of propidium iodide (PI) for 30 min at room temperature in the dark. Stained cells were analyzed by flow cytometry. For cleavage of PARP-1, cellular lysates were analyzed by Western blotting using anti-PARP-1 antibody as described below.

### 3.5. Western Blotting

Cell lysates were prepared using NP 40 cell lysis buffer. Lysates were clarified by centrifugation at 14,000 ×*g* for 10 min at 4 °C and protein concentrations were determined. Samples (50 μg) were boiled in an equal volume of sample buffer (20% glycerol, 4% SDS, 0.2% bromophenol blue, 125 mM Tris-HCl (pH 7.5), and 640 mM 2-mercaptoethanol) and separated on pre-casted Tris-glycine polyacrylamide gels using the XCell Surelock^TM^ Mini-Cell, in Tris-Glycine SDS running buffer, all from Novex (Invitrogen, Carlsbad, CA, USA). Proteins resolved on the gels were transferred to PVDF membranes. Membranes were blocked with 5% milk in 10 mM Tris-HCl (pH 8.0), 150 mM NaCl with 0.05% Tween 20 (TPBS) and probed using specific antibodies against proteins of interest or β-actin (loading control) and HRP-conjugated secondary antibody. Immune complexes were visualized with enhanced chemiluminescence. Protein bands were imaged and band densities analyzed using the NIH/Scion image analysis software. The protein band densities were normalized to the corresponding β-actin band densities and percent change in signal strength was calculated.

### 3.6. Measurement of hTERT Expression

The effect of CDDO-Me on hTERT expression was measured by analyzing hTERT mRNA and hTERT protein. For hTERT mRNA, total cellular RNA was extracted with TRI-zol reagent (GIBCO) according to the manufacturer’s recommendation. 1 μg of RNA was then reverse transcribed by oligo-dt primer and high fidelity reverse transcriptase (Boehringer, Mannheim, Germany) to generate cDNAs. One μL of cDNA was used as template for the polymerase chain reaction (PCR) using hTERT primers: upper, 5′-TGTTTCTGGATTTGCAGGTG-3′, and lower, 5′-GTTCTTGGCTTTCAGGATGG-3′; and GAPDH primers: upper, 5′-TCC CTC AAG, ATT, GTC AGC AA-3′, and lower, 5′-AGA TCC ACA ACG GAT ACA TT-3′. The PCR conditions used were 33 cycles of denaturation (95 °C for 1 min), annealing (62 °C for 30 s), and polymerization (72 °C for 1 min). The PCR products were separated on 2% agarose gel electrophoresis and visualized by ethidium bromide staining. Gels were photographed and band densities were analyzed using the NIH/Scion image analysis software. The hTERT primers amplified a DNA fragment of 200 bp and the DNA fragment size amplified by GAPDH primers was 173 bp.

### 3.7. Telomerase Activity Assay

The telomerase activity in cell extracts was assessed by the PCR-based telomeric repeat amplification protocol (TRAP) using TRAPeze gel-based telomerase detection kit from Millipore (Temecula, CA, USA) following manufacturer’s protocol. Briefly, cells were extracted in CHAP lysis buffer on ice for 30 min. Two μL (100 ng) of cell extract was added to the TRAP reaction mixture containing dNTPs, TS primer, TRAP primers and Taq polymerase and incubated at 30 °C for 30 min in a thermocycler followed by 3-step PCR at 94 °C/30 s, 59 °C/30 s, and 72 °C/1 min for 33 cycles. The PCR products were fractionated on nondenaturing 12.5% polyacrilamide gel and visualized by staining with ethidium bromide. The ladder of products with 6 base pair increment indicating telomerase activity was analyzed with NIH/Scion image analysis software. For silencing of hTERT, cells were transfected with double stranded siRNA-hTERT or non-targeting siRNA sequence using SignalSilence siRNA kit (Cell Signaling Technology, Beverly, MA, USA). Briefly, 2 × 10^6^ cancer cells were plated in 60 mm Petri dish for 24 h and treated with 3 mL of transfection medium containing 20 μg LipofectAMINE and 100 nM siRNA for 48 h. hTERT expression was analyzed by immunoblotting. For overexpression of hTERT, semi-confluent cell cultures were transfected with 10 μg of empty or hTERT expression plasmid (pCIneo-hTERT) using LipofectAMINE Plus reagent. After transfection for 48 h, cells were analyzed for the expression of hTERT by immunoblotting.

### 3.8. Histology

Specimens of the dorsolateral lobe of prostate gland (DLP) were fixed in 10% neutral buffered formalin for 48 h and then embedded in paraffin. Five micrometer thick sections were cut and stained with H&E for histology. Prostate lesions in the DLP were histologically graded as normal (ducts lined with single layer of secretory epithelial cells surrounded by 2–3 cell layers of fibromuscular stroma; low-grade PIN (epithelial cells with variably elongated nuclei with condensed chromatin); high-grade PIN (epithelial stratification and tufting, presence of micropapillary and cribiform structures); well differentiated (WD) carcinoma (epithelial cells invading fibromuscular stroma) and moderately (MD) to poorly differentiated (PD) adenocarcinoma of the prostate (sheets of neoplastic cells with little or no glandular structures). Ten randomly selected microscopic fields were scored for the incidence and pathologic grade of the prostate cancer in control and CDDO-Me-treated TRAMP mice.

### 3.9. Statistical Analysis

Most data are presented as means ± S.D. and outcomes for treated and untreated cells were compared by Student’s *t*-test. Differences were considered significant at *p* < 0.05.

## 4. Conclusions

This study has shown that inhibition of proliferation and induction of apoptosis in prostate cancer cells by CDDO-Me involves the inhibition of hTERT expression, protein phosphorylation and telomerase activity. CDDO-Me also inhibited hTERT regulatory proteins such as c-Myc, Sp1, NF-κB, p-STAT-3 and p-Akt in prostate cancer cell lines. Furthermore, the inhibition of prostate cancer progression in TRAMP mice by CDDO-Me was associated with the inhibition of TERT phosphorylation and TERT regulatory proteins in the prostate. Thus, targeting telomerase with CDDO-Me is a promising strategy for treating and/or preventing the prostate cancer.
